# This Rash Puts You in the ICU

**DOI:** 10.5811/cpcem.2018.9.39335

**Published:** 2018-10-17

**Authors:** Zachary Skaggs, Jordana Haber

**Affiliations:** University of Nevada, Las Vegas School of Medicine, Department of Emergency Medicine, Las Vegas, Nevada

## CASE PRESENTATION

**Image 1:**
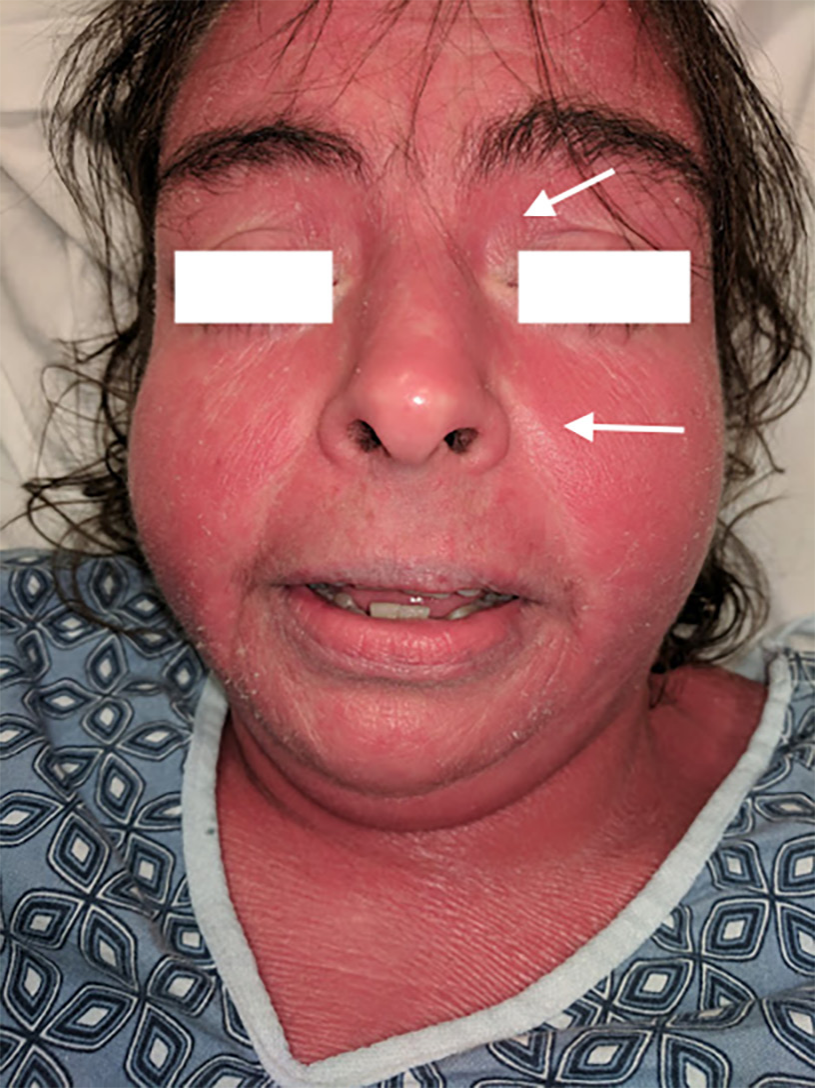
Erythroderma flare facial rash notably spares the periorbital area and nasolabial folds (arrows).

**Image 2:**
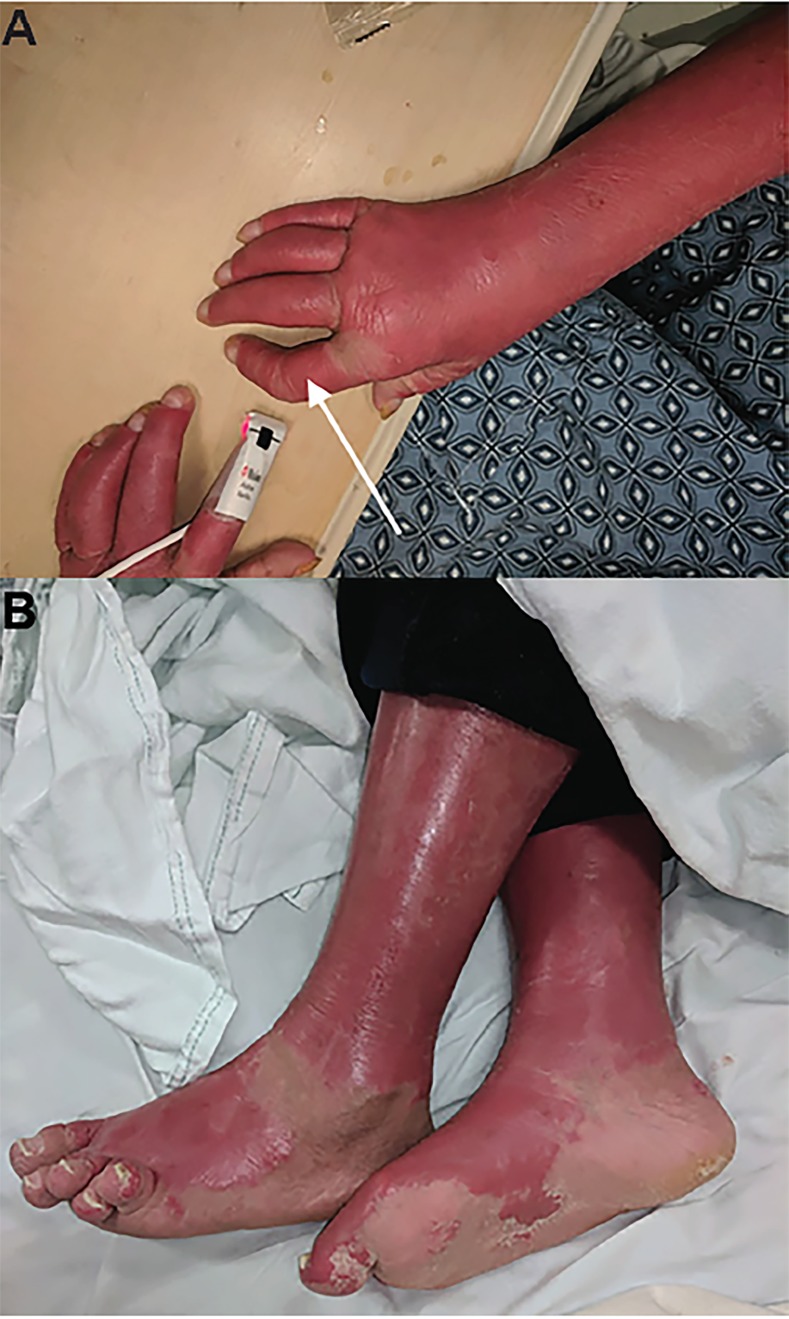
Erythrodermic psoriasis flare to the upper (A) and lower (B) extremities demonstrating dactylitis and Dupuytren’s contracture (arrow) consistent with long-term psoriatic arthritis.

A 33-year-old female with a history of psoriasis presented to the emergency department with a diffuse, pruritic skin rash that had been progressive for two days. She complained of associated subjective fever, chills, and myalgias. Her exam revealed a diffuse erythematous, blanching, non-tender rash to the face, body, and extremities (Images 1 and 2). The rash did not involve mucus membranes, but there was involvement of the palms and soles. There was scaling over the extensor surfaces and sparing of the flexor surfaces. The patient had been admitted to the hospital several weeks prior for a similar rash requiring intensive care unit (ICU) admission, steroids, and methotrexate.

## DIAGNOSIS

### Erythroderma (erythrodermic psoriasis flare)

Erythroderma, or “red skin,” is a severe cutaneous condition that presents with diffuse erythema involving greater than 75% of the body’s surface and skin exfoliation.^1^ It classically spares the periorbital regions and nasolabial folds. This patient also demonstrates findings consistent with long-term psoriatic arthritis such as Dupuytren’s contractures and dactylitis.^2^,^3^ While a prior visit required ICU admission, during this visit her vital signs were normal and she had an uneventful hospital course after the resumption of corticosteroid therapy.

**CPC-EM Capsule**

What do we already know about this clinical entity?

The majority of cases of erythroderma are secondary to exacerbation of a prior skin condition such as psoriasis. Important triggers include recent withdrawal of corticosteroid or antipsoriatic therapies, infection, and stress. Patients experience impaired temperature regulation and increased insensible losses. Increased skin breakdown makes these patients prone to superimposed cutaneous infections.

What is the major impact of the image(s)?

These images should aid in physician recognition of a potentially life-threatening rash. Patients with severe erythroderma flare as depicted here require supportive care in the form of temperature monitoring, fluid resuscitation, and electrolyte repletion. In severe cases, these patients may require admission to a burn care unit.

How might this improve emergency medicine practice?

Appropriate recognition of, management, and disposition of patient’s suffering from erythrodermic psoriasis flare.

Documented patient informed consent and/or Institutional Review Board approval has been obtained and filed for publication of this case report.
